# The Ecological Task Dynamics of Learning and Transfer in Coordinated Rhythmic Movement

**DOI:** 10.3389/fnhum.2021.718829

**Published:** 2021-09-07

**Authors:** Daniel Leach, Zoe Kolokotroni, Andrew D. Wilson

**Affiliations:** Psychology, Leeds School of Social Sciences, Leeds Beckett University, Leeds, United Kingdom

**Keywords:** learning, transfer of learning, bimanual coordination, ecological task dynamics, perceptual information for action

## Abstract

Research spanning 100 years has revealed that learning a novel perception-action task is remarkably task-specific. With only a few exceptions, transfer is typically very small, even with seemingly small changes to the task. This fact has remained surprising given previous attempts to formalise the notion of what a task is, which have been dominated by common-sense divisions of tasks into parts. This article lays out an ecologically grounded alternative, *ecological task dynamics*, which provides us with tools to formally define tasks as experience from the first-person perspective of the learner. We explain this approach using data from a learning and transfer experiment using bimanual coordinated rhythmic movement as the task, and acquiring a novel coordination as the goal of learning. 10 participants were extensively trained to perform 60° mean relative phase; this learning transferred to 30° and 90°, against predictions derived from our previous work. We use recent developments in the formal model of the task to guide interpretation of the learning and transfer results.

## Introduction

This article is the second part of a series of studies designed to investigate the perception-action mechanisms supporting learning and transfer of learning in coordinated rhythmic movement. Leach et al. (2021) extensively trained 10 participants to produce 90° mean relative phase using coordination feedback (Wilson et al., 2010b). As has been shown before (e.g., Wilson and Bingham, 2008) this training drove participants to stop trying to perceive relative phase with relative direction, and instead to use relative position. We then tested for transfer of this learning, and for the first time found substantial transfer to two other relative phases (60° and 120°). We explained the transfer as being supported by the use of relative position. In this article, we replicated the design of Leach et al. (2021) but now trained participants on 60°. We identified that this also led to them switching information variables to relative position, but contrary to our predictions, this time the learning supported transfer to 30° and 90°.

We will first review the issue of learning and transfer, and the ecological task dynamical analysis we use to understand these results. We will then review Leach et al. (2021) in more detail, to motivate the hypotheses and design of the current study. We will end by considering the implications of our results for ongoing attempts to model learning and performance in this task (Bingham, 2001, 2004a,b; Snapp-Childs et al., 2011; Hearth et al., under review).

### Learning and Transfer

One way to study the mechanisms of learning is to examine what else improves after training on some task. If learning one skill improves another, then those two skills must have something important in common that was affected by the learning process. *Transfer of learning* can therefore help us identify the components being brought together to perform these tasks.

Strangely, however, the data from hundreds of studies clearly show that learning rarely transfers in any meaningful way beyond the trained task; any observed transfer is typically small in magnitude. Seemingly small changes in the task requirements can block transfer; for example, training on a pursuit motor task at one speed shows little transfer to the same task at different speeds (Lordahl and Archer, 1958; Namikas and Archer, 1960). Even tasks that seem to have large overlap in the required components can show little transfer; for example, balancing on a slack line does not transfer to balancing on a beam, and vice versa (Serrien et al., 2017). The only way to observe large transfer is to keep the goal the same, but alter the performance requirements (e.g., training a coordinated rhythmic movement with the arms, testing for transfer using the legs, e.g., Kelso and Zanone, 2002).

This pattern is surprising given our typical understanding of what a “task” is and what is required to solve it. The change in speed in the pursuit motor task seems such a small change, but it has such a large effect. Walking on both slacklines and beams seems to require balance, but apparently “balance” is not a single element that can be deployed for different tasks. The data are clear; our current understanding of what makes something “a task” is flawed, and we need a better way. So what is the field currently doing, and what must we do differently?

Learning and transfer as a topic has swung in and out of fashion several times since the earliest experimental work by Thorndike and Woodworth at the turn of the 20th century. The basic logic has remained the same – transfer is expected to occur to the degree that two tasks share common elements. But each time, researchers have conceptualised tasks with intuition grounded in every-day language (e.g., walking on a slack line and a beam both require balance, so balance must be a key component of performance in both tasks) and each time, research has revealed little if any transfer between tasks described this way (for a detailed review, see Schmidt and Young, 1986; Perkins and Salomon, 1992). This intuition-based method is failing to carve nature at the joints. If we are going to break the boom-and-bust research cycle, we need a theory of tasks that is different in kind from the intuition-based theories that have come before. We need an empirically based theory of what tasks look like, from the first person-perspective of the organism.

### Ecological Task Dynamics

The ecological approach to perception-action (Gibson, 1979) is a theory of skilled action and learning that proposes a mechanism^1^ for how those come about. It characterises the world-to-be-perceived in dynamical terms; objects and events have properties that require units of time, position (and its temporal derivatives) and mass to characterise completely (Bingham, 1988, 1995). Dynamical systems behave when a particular set of these properties are coupled together, and the specifics of the behaviour depend on the composition but also the organisation of the properties; how they are coupled together.

Organism behaviours depend on dynamical properties of the organism, but also of the environment. These properties must be coupled together into a system with a specific composition and organisation for a particular behaviour to emerge from that system. That coupling is, in general, informational, because we are only in mechanical contact with a small fraction of our environment. Gibson proposed, and experimental evidence has confirmed, that perceptual information consists of higher-order invariant information variables in ambient energy media (e.g., the optic array). These variables can specify (map 1:1 to) dynamical properties of the environment (Turvey et al., 1981; Runeson and Frykholm, 1983). Organisms can therefore, in principle, learn to use that information to couple their own bodily dynamics to the behaviourally relevant properties of the environment, and a given behaviour emerges from this particular distributed dynamical system as it plays out over time and space.

There are three implications for learning and transfer in this analysis. First, learning a behaviour entails coupling dynamical properties distributed across the organism and environment via information, and the form of the resulting behaviour emerges from the composition and organisation of the entire organism-environment system, and not just the organism. Learning will therefore only transfer to the extent that the two organism-environment systems share that composition and organisation. Second, changing the organism dynamics but keeping the environmental dynamics the same allows the same information variables to be used in the coupling. This describes the case such as interlimb transfer tasks, where the goal remains the same but the performance requirements change, and as noted above, large transfer of learning is typically observed in these cases. Third, keeping the organism dynamics the same but changing the environmental dynamics means that the information that was previously available is no longer there; it is replaced by information specifying the new environmental dynamics. This describes the case such as the “balancing” experiment (Serrien et al., 2017) and, as noted above, these cases typically produce little or no transfer.

We can therefore put forward a hypothesis. If the dynamical properties within the organism-environment system are changed so that the informational coupling between the organism and environment is changed, this creates a new task dynamic and learning will not transfer. Information defines the boundaries of tasks. This specific application of task dynamics to perception-action systems is called *ecological task dynamics*.

### Testing Ecological Task Dynamics

Ecological task dynamics is a new way of characterising tasks that is based on the ecological analysis of behaviour, rather than everyday-language based intuitions about what we do. The proof of the pudding is in the tasting, of course; so can this new notion of task do better than the old in accounting for patterns of learning and transfer?

The ecological task dynamics analysis has so far been developed most completely in the context of coordinated rhythmic movement (e.g., Kelso, 1995). This task asks people to rhythmically move two limbs so as to produce a target mean relative phase (or sometimes to move one limb at some mean relative phase to an oscillator on a monitor). The key phenomena to be explained are that (a) people can readily produce and maintain 0° and 180° at a range of frequencies, although at high frequencies people tend to transition from 180° to 0° without a lot of effort, and (b) other mean relative phases (especially the intermediate 90°) must be learned using some form of feedback.

This task has been formalised in a perception-action task dynamical model (Bingham, 2001, 2004a,b; Snapp-Childs et al., 2011, 2015). This mechanistic model implements the real dynamical properties of the limbs (as phase-driven, nonlinear damped mass springs) and the real informational coupling between them (perceived relative phase, modelled as the relative direction of motion, conditioned on the relative speed). All of the dynamical and information components of the model have been empirically verified to be part of the perception-action system from which coordinated rhythmic movement behaviour emerges (see Golonka and Wilson, 2012 for a review) and the model reproduces all the key phenomena of the task described above. Most relevantly for this paper, these phenomena are caused by the relative direction informational coupling.

Learning experiments have shown that people can improve at 90°, and that they do so by learning to perceive relative phase using relative position instead of relative direction. For example, Wilson et al. (2010a) trained participants to improve their performance at 90° by improving their visual discrimination of 90° movements. Wilson and Bingham (2008) then selectively perturbed candidate information variables and showed that performance at 90° was only fully disrupted by a perturbation of relative position.

There are then two transfer studies relevant to the current paper. The first was Snapp-Childs et al. (2015), who trained participants at 90° on either the unimanual or the bimanual version of the coordination task. Participants moved one or two joysticks so as to produce 90° between two dots on a computer monitor (in the unimanual case, the computer controlled one dot). Participants improved in each training group, and that training then transferred quite significantly between the conditions (proportion transfer was 43–45%). Improvement in performing 90° also transferred to improvements in visual discrimination of 90°. This experiment altered the organism dynamics via training group (i.e., number of limbs) and the informational coupling via the training (switching from relative direction to relative position). But because both versions of the task entailed the same informational coupling at the end of training (relative position) there was transfer between them at the end. Both the unimanual version of the task (Snapp-Childs et al., 2011) and the bimanual version of the task (Bingham, 2001, 2004a,b) have been modelled using the same informational coupling.

Most recently, and directly preceding the current study, Leach et al. (2021) extensively trained 10 participants to produce bimanual 90°. We tested their performance of 0°, 30°, 60°, 90°, 120°, 150°, and 180° and their visual perceptual thresholds for 90° in three Assessment sessions (Baseline, Post-Training, Retention). In the two post training sessions, we also tested their visual perceptual thresholds for 90° under the perturbation of relative position. We found the following main results:

1. All participants learned to produce 90° well, and this was accompanied by a decrease in their visual thresholds for discriminating 90° (replicating Snapp-Childs et al., 2015). Post-training, the improvement in the visual discrimination of 90° was entirely wiped out by the position perturbation, confirming that the participants had improved at 90° by learning to use relative position (replicating Wilson and Bingham, 2008).
2. Learning to produce 90° transferred substantially but asymmetrically to 60° and 120°. We observed a massive 81% transfer to 60°, and a still large 65% transfer to 120°. (We explained the asymmetry as the result of relative speed still acting as a noise term on the perception of relative phase; relative speed increases linearly from 0° to 180°; Snapp-Childs et al., 2011).

Overall, the results of these two transfer studies support the dynamical analysis of what a perception-action task is, from the first-person perspective of the organism. Learning alters the overall task dynamic of the system producing the behaviour, specifically in this case by altering the informational coupling between the limbs. We know that learning transfers to other versions of the task that entail different limb dynamics but can still use the new information coupling (e.g., between the unimanual and bimanual versions of the task); we therefore explained the results of Leach et al by hypothesising only 60° and 120° could be produced using relative position to couple the limbs.

The current experiment is the next step in understanding what happens to the perception-action dynamics responsible for coordinated rhythmic movement after learning. We exactly replicated the design of Leach et al. (2021), but this time we trained participants to produce bimanual 60° and examined the pattern of transfer across six other relative phases. Based on our understanding of the changes in the dynamics so far, we made the following predictions:

1. Participants would be able to learn 60°, and this would entail a switch to using relative position to perceive relative phase. This is tested using the perturbation method, whereby perturbing relative position in a two forced alternative judgement task will completely disrupt trained performance.
2. We know that only 60°, 90°, and 120° have been shown to benefit from using relative position to perceive relative phase. We therefore predicted that learning to perform 60° would transfer only to 90° and 120°, with the proportion of transfer conditioned by the relative speed^2^.

## Materials and Methods

This experiment’s design and analysis plan was preregistered (Leach et al., 2017). Sample size (*N* = 10) was justified with a power analysis in which the results of Leach et al. (2021) informed the expected transfer effect sizes (see section “Appendix 1”).

### Participants

Eleven adults participated in this study, one of whom chose not to complete the entire procedure leaving a total of 10 participants (19–33 years old, *M* = 26.1; Male = 3, Female = 7). All participants were free from known neurological defects or motor disabilities, had normal or corrected-to-normal vision and were right-handed (measured with the Edinburgh Handedness Inventory; Oldfield, 1971; Dragovic, 2004). All participants were naïve to the experimental questions. Prior to training, all participant’s relative phase production matched the predefined criterion for participation (see section “Criteria”). All participants were recruited using a convenience sample in the surrounding area of Leeds, United Kingdom and paid £15 upon competition of the study. Ethical approval was granted by the Psychology Ethics Committee at Leeds Beckett University, United Kingdom.

### Design

The design was identical to that used in Leach et al. (2021) except that the target relative phase being trained was now 60°. All participants performed two types of experimental task; coordinated rhythmic movements (*Action*) and two-alternative forced choice (*Judgements*).

For the Action tasks, there were two within-subject variables. The first is Session (three levels; Baseline, Post Training, and Retention). These sessions were referred to as Assessment sessions, to distinguish them from the Training sessions. The second was Target Phase (seven levels; 0°, 30°, 60°, 90°, 120°, 150°, and 180°). The dependent variable was the Proportion of Time on Target phase ± 20° (PTT20), a valid measure of performance (see Snapp-Childs et al., 2011, 2015 for explicit comparisons of this to other commonly used measures, which motivates us to prefer PTT20).

For Judgement tasks, there was one within-subjects variable, Session (three levels; Baseline, Post Training, and Retention). The dependent variable was the estimated Threshold to identify 60° in the Judgement tasks (the lower the threshold, the greater the ability to discriminate 60°).

### Materials

All sessions were performed on a “Windows PC with a 24” Dell monitor located approximately 70 cm from the participants. The computer presented a display of two white dots (∼15 mm), separated vertically (∼35 mm), that moved horizontally across a black background (screen refresh rate 60 Hz, resolution 1,920 × 1,080). The motion of both dots was centred at the screen centre with an amplitude of 300 pixels (∼115 mm). All displays were presented, controlled and recorded by a custom MATLAB toolbox written by ADW incorporating the Pyschtoolbox (Brainard, 1997; Pelli, 1997; Kleiner et al., 2007; http://psychtoolbox.org). Matlab 2014b was used to record and analyse the data.

**FIGURE 1:**
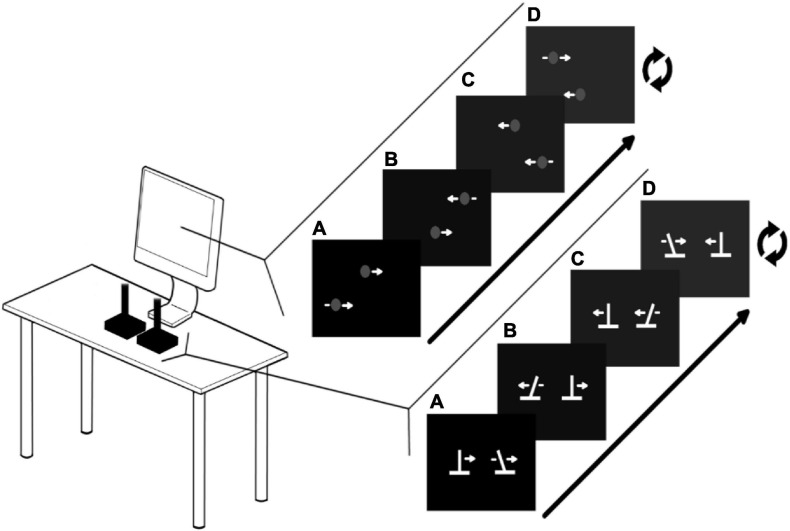
Experimental setup : Action sessions. Participants use both joysticks to control the horizontal movements of the dots on the computer display. The visual display on the computer screen (A) corresponds with the position of the joysticks (A). The figure shows an example of moving at 60°. This is achieved by moving linearly from A to D and repeating. During the training sessions, moving at 60° ± some error triggers the hot-cold signal in which the white dots turn green (grey in figure; see Coordination Feedback).

For Action sessions, participants used two USB Logitech Extreme 3D Pro joysticks. The central spring and the rubber guard were removed to disable force feedback (see Figure 1). The vertical position of both dots on the screen was fixed, but the horizontal position of both dots were controlled by the horizontal position of the joysticks, with the left and right joystick corresponding to the top and bottom dots, respectively. The mapping of the joysticks to screen amplitude is set so that required amplitude on the screen does not entail hitting the limits of the joystick range of movement. This forces participants to actively control the joysticks as much as possible, rather than to simply slam into the joystick endpoint to stop.

For Judgement sessions, the participant responded to displays using a USB keyboard. Responding with the “A” and “L” keys for the first and second choice, respectively.

### Procedure

Participants performed between nine and thirteen separate sessions on separate days (see Table 1). The exact number of sessions performed by each individual participant was dependent on when various criterion were met during training (see section “Criteria”). During the Baseline assessment session, participants performed three different tasks (two Action, one Judgement) in the order described (approximately 45 min to complete). In the Post-Training and Retention assessment sessions, participants repeated the procedure from Baseline with one additional perturbation Judgement session described below (approximately 60 min to complete). Participants completed the Baseline, Training and Post-training sessions within a 3 week time frame, and completed the Retention session 14–24 days after the Post-Training session. Each Training session took approximately 20 min to complete.

**TABLE 1:** Experimental design.

**Baseline** *1 session*	5× 20 s trials each of bimanual 0°, 180°, *assigned phase* (60°) *Criterion for participation: 60°* < 0*° and 180°; assigned phase (60°) < 0.50* 5× 20 s trials each of bimanual 30°, 90°, 120°, 150°
	2AFC judgement task (*assigned phase*, 60°)
**Training**	30× 20 s trials bimanual 60° w/feedback ± 30°
	30× 20 s trials bimanual 60° w/feedback ± 25°
*6–10 sessions*	30× 20 s trials bimanual 60° w/feedback ± 20°
	30× 20 s trials bimanual 60° w/feedback ± 15°
	30× 20 s trials bimanual 60° w/feedback ± 10° 30× 20 s trials bimanual 60° w/feedback ± 10°
**Post training**	5× 20 s trials each of bimanual 0°, 180°, *assigned phase* (60°) 5× 20 s trials each of bimanual 30°, 90°, 120°, 150°
*1 session*	2AFC judgement task (*assigned phase*, 60°) 2AFC judgement task (Perturb Position)
**Retention** *1 session*	5× 20 s trials each of bimanual 0°, 180°, 60° 5× 20 s trials each of bimanual 30°, 90°, 120°, 150° 2AFC judgement task (*assigned phase*, 60°) 2AFC judgement task (Perturb Position)

*All participants worked through these tasks in the order noted. The feedback bandwidth (e.g., ±30°) indicates over what range from the target-phase the colour feedback is triggered. This is faded over time to drive learning (Wilson et al., 2010a,b). See Criteria regarding the performance-based progression employed.*

### Action Task (Assessment Sessions)

All participants were shown an 8 s, 1 Hz demonstration of the first target relative phase (0°) and performed one 20 s practice trial of producing that relative phase at 1 Hz with the joysticks. Participants then performed one block of four 20 s trials in which they controlled the horizontal motion of both dots. The top dot was controlled by the left hand, the bottom dot by the right hand. Participants were instructed to move the joysticks in a smooth, side-to-side, movement to produce the target-phase at 1 Hz. This block structure was then repeated for 180° and 60° relative phase, in that order.

These data were used to ensure that none of the participants were already able to perform 60° at a level equivalent to 0° and 180° and could take part in the study (see section “Criteria”). After this, participants performed a second set of coordinated rhythmic movements to measure baseline performance at 30°, 90°, 120°, and 150°, using the same structure as above.

### Judgement Task

Following the action tasks, participants performed a series of two-alternative forced choice (2AFC) judgements for 60°. 2AFC is a standardised psychophysical measure for determining perceptual thresholds (see Wilson and Bingham, 2008; Wilson et al., 2010a; Snapp-Childs et al., 2015 for applications to coordination perception).

Each trial started with a 4 s demonstration trial of 60°, followed by the presentation of a pair of successive displays. Both displays contained two dots moving harmonically on the screen at some mean relative phase, for 4 s at 1 Hz. The dots were centred on the screen, with an amplitude of 300 pixels (∼11.5 cm). Of each pair, one showed two dots moving at 60°, and the other was different from 60°; the order was randomly selected on each trial. The task for the participants is to choose which one of the displays shows 60° (pressing “A” for the first and “L” for the second, with no speed requirement).

How different the two displays were was determined using two independent but interleaved transformed 1-up/2-down staircase procedures. One staircase controlled the different displays less than 60°, one for those greater than 60°. Both used a step size “up” of 10° and a stop rule of 8 reversals. Step size “down” was fixed to 54.88% of the step size “up” according to **Table 5**.1 of Kingdom and Prins (2009); here 5.48°. The initial difference for each staircase was set to 30° and trials only stepped down until the first reversal (first error), after which the staircase procedure was applied. Participants are given knowledge of results (KR) after each trial (“Correct!” or “Incorrect!”). This procedure is essentially identical to that used in Snapp-Childs et al. (2015) with the addition of the KR.

In the Post Training and Retention sessions, participants repeated the 2AFC task and then completed an additional 2AFC task in which a position perturbation is applied to the display (Wilson and Bingham, 2008). In these displays, the amplitude of the top dot is changed at random on every half cycle, with the constraint that the dot must cross the midline of the screen and cannot exit the screen. The amplitude of the bottom dot is then set to half the top dot’s amplitude, so that it varies randomly but in a way that is coupled to the other dot – this preserves the relative phase. Where and when peak amplitude and peak velocity occur therefore change on every half cycle. This preserves mean relative phase (and relative direction information about that relative phase) while making it impossible to use relative position information to perceive relative phase, because there is no stable information about where the dots are within their cycles. This perturbation tests the hypothesis that learning to improve at 60° entails switching to using relative position.

### Action Task (Training)

Following Baseline assessment, participants were trained to bimanually produce 60°. The number of training sessions completed by each participant depended on their performance (see section “Criteria”). The number of training sessions across participants varied between 6 and 10.

During each training session, participants performed thirty 20 s trials where their goal was to produce 60°. Participants received *coordination feedback* for all trials except for every fifth trial (Wilson et al., 2010b). This feedback changed the colour of the dots from white to green when performance was within the given error bandwidth of the target relative phase. In the first training session the error bandwidth is set at ±30° and was reduced by ±5° across sessions when the Criterion for Progression was met (to ±25°, ±20°, ±15°, and ±10°). This colour feedback was not present in the Assessment Action tasks; or that reason, coordination feedback is removed every fifth trial to help prevent dependence on it (Kovacs et al., 2009; Snapp-Childs et al., 2015).

After every trial with feedback participants also received KR feedback based on their performance, in which the participant is given a performance percentage (their PTT20 score as a percentage) and a comment (see Table 2). Finally, participants received additional KR at the end of each training session in the form of a level-progression statement. This simply stated whether or not the participant would stay at the current level or progress to the next level. We found that this helped participants stay on task and remain motivated through the extensive training.

**TABLE 2:** Knowledge of results (performance generated score).

**Performance**	**Comment**
<25%	*=This is still a little low – keep trying!*
25–50%	*=Definitely improving – keep it up!*
50–75%	*=Doing great – keep it up!*
>75%	*=This is really great – great job!*

#### Criteria

Prior to training, all participants’ 60° production was substantially worse than 0° and 180° (Mean PTT20: 0.22; 0.77; 0.81, respectively). Participants were then trained in accordance with several pre-defined criteria (see preregistration). In each training session, when PTT20 was greater than 0.5 in at least 20/30 trials, the participant progressed to the next training stage. This was used to confirm that the participant was ready for progression and to avoid occasional poor performance trials from halting progression. Meeting this criterion resulted in the feedback bandwidth of the next training session to be reduced by ±5°; otherwise the feedback was kept the same. Training was stopped if PTT20 was greater than 0.6 in at least 20 trials for the last two training sessions (feedback bandwidth at ±10°), or when participants completed 10 training sessions. Participants completed between 6 and 10 training sessions. All participants progressed to and completed at least one session with the feedback bandwidth set to ±10°.

### Data Analysis

#### Judgements

For the judgement tasks, the computer recorded the responses (“correct” or “incorrect”) in relation to the relative phase of the “different” displays that were shown. We separately averaged the difference from 60° of relative phases at which reversals in the staircase procedure occurred for the “different” phases that were greater than 60° and those less than 60°, excluding the first reversal, for each participant. We then averaged those thresholds for each participant.

#### Movement

The raw movement data is a 60 Hz time series of the position of the joysticks over time. Each time series was centred on 0, filtered with a low-pass Butterworth filter (cut-off frequency 10 Hz), and differentiated to compute the velocity time series. The continuous phase time-series of each joystick was computed as the arctan(V/X) for each data point and the difference between these time series was the relative phase time series. We then computed the proportion of this time series that fell within 20° of the target relative phase (PTT20).

#### Contrast Analyses

To analyse transfer of learning we used Dependent Measures Contrast Analyses (Rosenthal et al., 2000). This analysis allows us to test for a specific hypothesised pattern of differences across multiple means with a single test (rather than the less powerful and less targeted method of an ANOVA followed by pairwise comparisons). In this experiment, we applied a contrast analysis to performance in the three Assessment sessions at each untrained relative phase in which we tested for the specific pattern of change observed at the trained relative phase of 60°.

The test statistic, *t*, is computed as

(1) $$t_{\text{contrast}}=\frac{\bar{L}}{\sqrt{\frac{{\hat{\sigma }}_{L}^{2}}{n}}}\mathrm{with}L_{i}=\sum_{j}^{k}\left(x_{ij}\cdot \lambda_{j}\right)$$

where *x* is the data and λ are weights. The λ weights are the way of quantifying the hypothesised pattern, here set by Assessment session performance at 60° (see below). If the data do not differ in the specific way implemented by the Lambda weights (λ), then *L*_*i*_ is near to zero (i.e., H_0_ is *L*_*j*_ = 0). In terms of transfer, a statistically significant *L*_*i*_ score for data at a particular untrained relative phase indicates that the specific pattern of improvement observed at 60° is also occurring at that particular untrained phase; the learning has transferred.

## Results

Performance was examined across Assessment sessions at 60° to identify whether and how participants had improved with the training. The identified pattern of learning was used to set the λ weights for the contrast analyses. These analyses provide the tools to investigate whether the observed pattern of learning at 60° had transferred to any of the other untrained relative phases (0°, 30°, 90°, 120°, 150°, and 180°). This basic analysis plan was then repeated with the Judgement data.

### Learning

**FIGURE 2:**
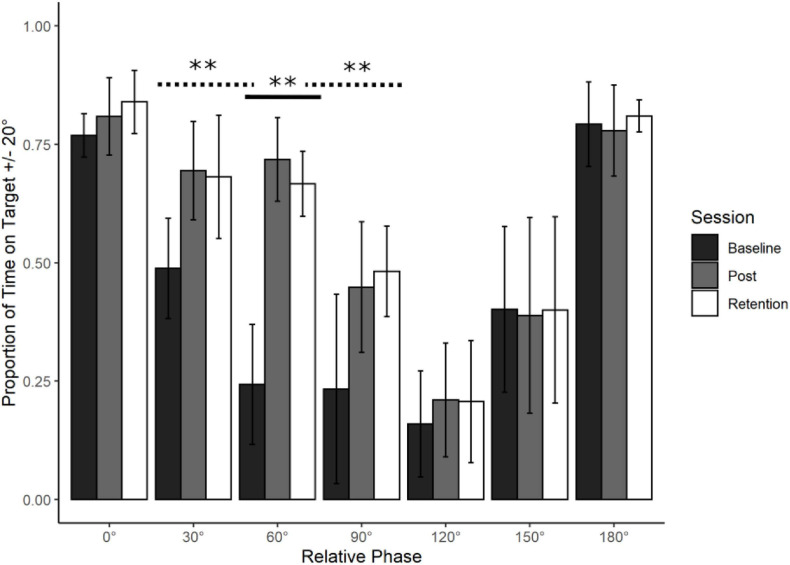
Young adults trained at 60° : Average action data. Average performance data (Proportion of Time on Target ± 20°) with standard error bars for all phases in the three assessment sessions (Baseline, Post Training, and Retention). Significance levels are indicated on the figure (^∗∗^*p* < 0.01). There was a significant main effect of Session for the trained phase of 60° (solid line). This learning transferred to 30° and 90° (dotted lines, see Transfer section for further detail).

Refer to Figure 2. To examine whether and how training at 60° changed performance at 60°, average PTT20 was analysed using a one-way repeated measures ANOVA with Session (Baseline, Post Training, and Retention) as a within-subject factor.

Participants significantly improved their coordination stability from Baseline to Post Training and that learning was Retained. There was a main effect of Session, *F*(2, 18) = 149.95, *p* < 0.001. Bonferroni-adjusted post-hoc analyses revealed a significant difference between Baseline and Post Training, *t*(9) = 15.78, *p* < 0.001, MD = 0.475, Baseline and Retention, *t*(9) = 14.068, *p* < 0.001, MD = 0.424 but not between Post and Retention *t*(9) = 1.712, *p* > 0.05, and MD = 0.052.

The observed learning pattern mirrored what happened in Leach et al. (2021). Production of 60° was poor at Baseline, it improved significantly with training and this improvement remained stable after the retention period. Using the learning pattern identified at 60°, the λ weights for the Action data were set at -2 for Baseline, 1 for Post Training and 1 for Retention. This was done in accordance with the guidelines set by Rosenthal et al. (2000).

### Transfer

Based on the results of Leach et al. (2021), we made explicit predictions regarding where transfer was likely to take place. Learning 90° entails a switch from relative direction to relative position. A consequence of this switch was improvement at 60° and 120°. If relative position affords stability at 60°, 90°, and 120° then learning to produce any of these three relative phases should induce transfer to the other two. Thus, the prediction is that learning 60° will transfer to 90° and 120°. There is no reason that transfer will occur at 0° or 180°, so these phases were not tested. Using the λ weights assigned from the learning data of 60° (-2, 1, and 1), Dependent Measures Contrast Analyses were completed across Assessment sessions for the four criterion tasks (30°, 90°, 120°, and 150°). Any significant results would indicate significant transfer, showing that the same pattern of learning at 60° was present in the criterion task. Any transfer was expected to be very large in effect (*Hedges g* > 1.5).

**FIGURE 3:**
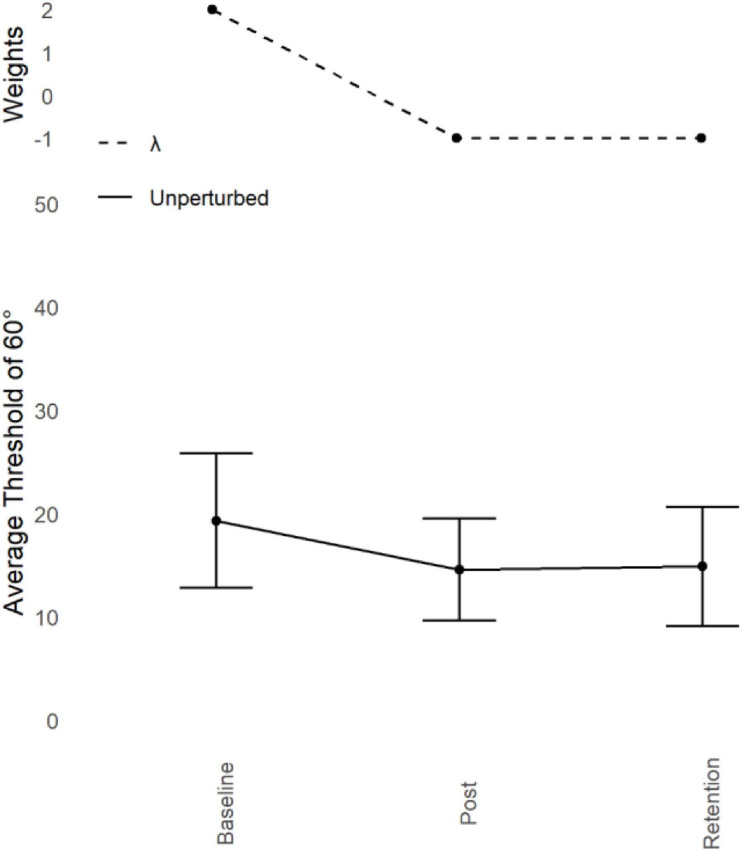
Young adults trained at 60° : Judgement transfer. Average unperturbed Perceptual Judgement Thresholds for 60° (lower) with standard error bars at Baseline, Post Training, and Retention with corresponding Lambda (λ) weights (upper). Average unperturbed thresholds reduced as a function of training over time, indicating an improvement in performance. This improvement remained after the retention period.

Refer to Figure 3. Contrary to our predictions, Dependent Measures Contrast Analyses with *Holm-Bonferroni* corrections revealed significant transfer to 90° *t*(9) = 4.476, *p* < 0.001, *g* = 1.415 and 30° *t*(9) = 4.402, *p* < 0.001, *g* = 1.39 but nowhere else (*p* > 0.05).

Proportion of transfer was calculated across all conditions by taking the difference between Post Training and Baseline performance for the criterion task and dividing that by the difference between the Post Training and Baseline performance for each of the transfer tasks. Performance at 150° (-3%) and 180° (-3%) was negligibly worse as a function of practice at 60°. All other phases increased in performance as a function of practice at 60°. There was some increase of performance at 120° (11%) and 0° (8%), but the only substantial increase was at 90° (45%) and 30° (43%). There was no structural change in the proportional transfer at retention, other than an increase in the transfer to 90° (58%) where all other phases stayed reasonably stable.

### Judgement Thresholds

**FIGURE 4:**
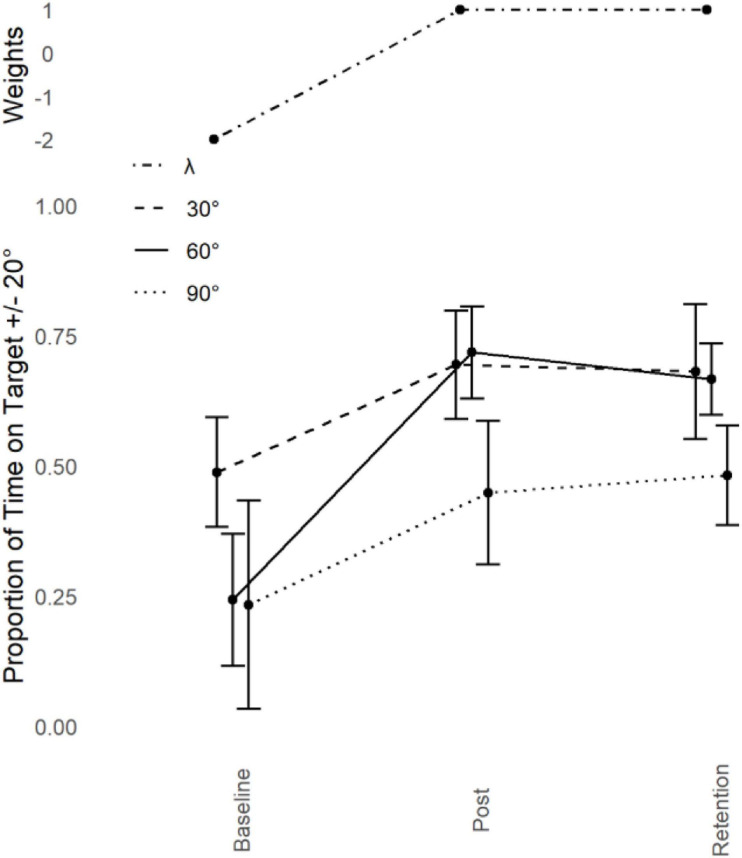
Young adults trained at 60°: Performance transfer. Average performance data (Proportion of Time on Target ± 20°, lower) with standard error bars (lower) for the trained phase of 60° and transfer partners 30° and 90° in the three assessment sessions (Baseline, Post Training, and Retention) with corresponding Lambda (λ) weights (upper).

Refer to Figure 4. Prior to training, thresholds for identifying which display showed 60° were high (*M* = 19.32°, *SD* = 6.53°). After training, this threshold improved (*M* = 14.58°, *SD* = 4.92°) and remained low after the Retention period (*M* = 14.89°, *SD* = 5.77°).

### Contrast Analyses

To test the prediction that the Judgement data mirrors the learning data in the Action task (60°) the λ weights identified in the learning pattern of 60° were used to predict the same pattern in the Judgement data. The lower the threshold, the greater the ability to discriminate between the target relative phase (60°) and other relative phases. Thus, the sign of the λ weights are reversed to comply with the nature of the measure (2 for Baseline, -1 for Post Training, and -1 for Retention). A Dependent Measures Contrast Analysis with the within subjects factor of Session (3 levels; Baseline, Post-Training, and Retention) and the dependent variable of unperturbed judgement thresholds of 60°, revealed a significant effect with a large effect size. Replicating experiment 1, this demonstrates that the Action-driven λ weights are a good fit for the Judgement data, *t*(9) = 4.152, *p* < 0.001, and *g* = 1.31.

### Unperturbed and Perturbed Judgement Threshold Comparison

**FIGURE 5:**
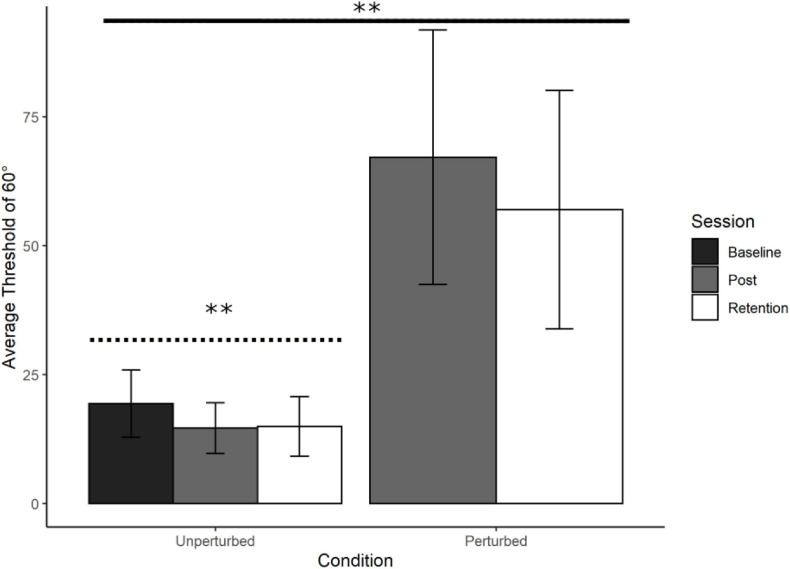
Young adults trained at 60°: Average perceptual judgement thresholds. Average perceptual judgement thresholds for 60° with standard error bars at Baseline, Post training and Retention. Significance levels are indicated on the figure (^∗∗^*p* < 0.01). There was a significant main effect of Condition, with the perturbation reducing performance (solid line). The contrast analysis demonstrated that the learning data was a good fit for the unperturbed judgement data (dotted line).

Refer to Figure 5. Thresholds for identifying 90° were lower than Baseline in the unperturbed condition in both Post Training (*M* = 14.58°, *SD* = 4.92°) and Retention (*M* = 14.89°, *SD* = 5.77°) but were extremely high and variable in the perturbed condition for both Post Training (*M* = 67.12°, *SD* = 24.63°) and Retention (*M* = 56.97, *SD* = 23.12). Participants improved perceiving and moving at 60° by switching to using relative position, and when this was no longer informative about relative phase, they could no longer perform the judgement task.

To compare the Unperturbed judgement thresholds at Post Training and Retention with the Perturbed judgement thresholds we performed an ANOVA on average judgement thresholds with Condition (Unperturbed and Perturbed) and Session (Post Training and Retention) as factors. There was a significant main effect of Condition, *F*(1, 18) = 49.71, *p* < 0.001, with no other significant main or interaction effects. The perturbation resulted in a substantially larger perceptual threshold at Post Training and Retention in comparison to the unperturbed condition (see Figure 5). The lower the threshold the better the performance.

### Bias Analysis

The dependent variable PTT20 (proportion of time on target within a tolerance range of 20°) is an established measure of assessing performance of the task over time (see Wilson et al., 2010a,b for initial use, and Snapp-Childs et al., 2015, for a detailed comparison with other measures). The standardised bandwidth of ±20° has repeatedly captured changes in performance over the course of learning, so it is reasonable that this experiment uses this bandwidth (for previous studies closely aligned to this one that also use this bandwidth, see Snapp-Childs et al., 2015; Leach et al., 2021).

As per Leach et al. (2021), during the early phases of training, the feedback was triggered over a wide range (±30) and this was then reduced according to performance. In addition, the ±20° bins for each neighbouring relative phase overlap. One potential issue is that what we have reported as transfer (say to 30° ± 20°) could simply be a bias toward one instance of the feedback or time spent moving within the 60° ± 20° bin. For example, when presented with the task of moving at 60° an individual might spend time moving at 45°, which is within our PTT20 threshold for “on target” for both 60° and 30°.

We repeated the transfer analysis with a reduced bandwidth of ±10°^3^. If bias toward a particular instance of the feedback is what is driving the transfer effect, then the results should be characteristically different. That is, the performance landscape should look different. However, if PTT20 is successfully capturing transfer, the results will replicate (likely with a reduced effect, as performing within ±10° requires a higher degree of accuracy).

As per Leach et al. (2021), the learning pattern found with a reduced bandwidth of PTT10 at 90° was identical to the PTT20 result. Participants improved their coordination stability from Baseline to Post Training and that learning was retained. An ANOVA confirmed this with a main effect of Session [*F* (2, 18) = 117.65, *p* < 0.001]. The difference between Baseline and the other Assessment sessions was driving this effect (both *p* < 0.001), and there was no significant difference between Post Training and Retention (*p* > 0.05).

As the learning pattern was the same at PTT10, the weights for the contrast analysis were set the same (-2, 1, and 1). The pattern of transfer found with the reduced bandwidth mirrors what was found at PTT20, with reduced effect sizes (yet still large in magnitude). The learning at 60° transferred to 30° [*t* (9) = 4.67, *p* < 0.001, and *g* = 1.48] and 90° [*t* (9) = 4.6, *p* < 0.001, *g* = 1.46], and nowhere else (*p* > 0.05).

This analysis tells us two things. First, the pattern of transfer we observed was not caused by any systematic bias in how participants performed during training. Second, it confirms that the 20° bandwidth for the PTT20 measure is appropriate; the bandwidth doesn’t dictate the pattern of results (see also Wilson et al., 2010a who checked bandwidths of 10°, 15°, and 30° and found the same result).

## Discussion

This study aimed to further probe the transfer effects found in Leach et al. (2021). There, learning to produce 90° transferred to 60° and 120° and this transfer was supported by the switch to using relative position to perceive relative phase. This study trained participants to produce 60°; we predicted improvement would come with a switch to relative position and a consequent transfer to 90° and 120°.

As predicted, learning to produce 60° improved the visual discrimination of 60°, and this was caused by a switch to relative position (demonstrated by the position perturbation results). We again saw transfer of learning to two different relative phases. However, the location of the transfer went against predictions. Transfer occurred at 30° and 90° and there was no asymmetry; transfer occurred in a proportionally symmetrical manner to both 30° (43%) and 90° (45%). The proportion of transfer was also smaller than in Leach et al. (2021).

We can rule out several possible explanations for the contrasting results. Firstly, we can rule out the idea that participants did not learn relative position at 60°, but some other information variable; the disruptive effect of the perturbation procedure on trained performance is clear. In the position perturbation, relative direction is unaffected and people using it are also unaffected (Wilson and Bingham, 2008). Therefore, participants are not using relative direction as information for learning 60°. Secondly, the smaller magnitude of transfer was not caused by less learning, which was comparable across 90° (0.43 improvement) and 60° (0.48 improvement). Baseline performance of 60° and 90° were also almost identical over both experiments.

### Does This Refute Ecological Task Dynamics?

At first glance, these results seem to expose a weakness in the ecological task dynamical approach. We made a prediction, and the results do not accord with that prediction. There is another, related literature on coordination dynamics that might accommodate these results; specifically, the dynamical systems account developed primarily by Zanone and Kelso (1994, 1997) and embodied in various versions of the Haken-Kelso-Bunz model (Haken et al., 1985). This account and the ecological task dynamical account are at odds with each other in various ways (Golonka and Wilson, 2019) and perhaps our results favour them over us.

This theoretical framework describes coordination dynamics in terms of attractor layouts, and models learning as a system wide phase transition from a bi-stable (0°, 180°) to a tri-stable arrangement of attractors (0°, 180° plus the trained relative phase). With regards to transfer, experiments tend to find that learning transfers to the symmetry partner of the trained relative phase (where the timing is the same but the lead-lag relations between the limbs is reversed, e.g., 90° and 270°). Less discussed is the fact that experiments often also show improvements in relative phases that neighbour the trained relative phase (e.g., see Figure 1a in Zanone and Kelso, 1997; Hurley and Lee, 2006, also saw transfer from 90° to 135° under one particular feedback condition). This could, in theory, be accounted for by the attractor formed by training being wider than the intrinsic attractors at 0° and 180°, meaning more neighbouring states might be included in the trained attractor.

However, this account has serious issues. There is strong evidence that the attractors are not real parts that are causing behaviours. For example, Zanone and Kelso (1994) predicted that attractor strength would affect learning rates, specifically that learning something close to 0° (e.g., 30°) would be harder than learning something the same distance from 180° (e.g., 150°) because the stronger attractor at 0° would compete with the to-be-learned pattern more fiercely. The opposite is actually true (Fontaine et al., 1997; Wenderoth et al., 2002). Second, a wide attractor is not an especially stable state, nor can it distinguish between the states within it. Our results (especially the bias analysis) show that people were actually moving at the transfer relative phases, and doing so quite well. Finally, with regards to applying these results to the current study, dynamical systems theory experiments use different feedback methods (either visual metronomes or Lissajous figures) and these displays alter the task dynamic by altering the information supporting the coupling, and this changes the overall behaviour of the system quite drastically (e.g., Kovacs et al., 2009, 2011).

So, while attractor dynamics is one legitimate way to describe the system dynamics, it is not an explanation of those system dynamics – there is no mechanism at work in those models (Golonka and Wilson, 2019). This account is effectively just a data-fitting exercise, and it does not help us understand our results. In order to try and develop an actual mechanistic explanation of our results, we will therefore continue to apply the ecological task dynamical approach and its real parts and processes (specifically, information variables and limb dynamics). This approach to defining a task is empirical, and the data are telling us that we do not yet have the right dynamical description of the two trained systems. We are missing a piece of the puzzle, and so while we do not yet know what that is, we can develop some hypotheses based on the ecological analysis.

### Expanding the Task Dynamical Analysis

Right now, the dynamical model of coordinated rhythmic movement (Bingham, 2001, 2004a,b; Snapp-Childs et al., 2011) is a model of the untrained system; the limb dynamics are coupled via relative phase perceived using relative direction. Over training, the limb dynamics remain the same; what changes is the perceptual coupling. Training 90° and 60° both lead to participants being sensitive to the position perturbation, but how they are using that information produces different patterns of transfer. The question is why.

The first option is that the position perturbation judgement task is not as specific as it was designed to be, and participants have in fact learned two different information variables that just happen to both get hit by the position perturbation. This is unlikely. The only other two candidates (relative speed and relative frequency) are both affected by the position perturbation, but Wilson and Bingham (2008) showed that perturbing these individually only added noise to the judgements. In addition, neither is robust information for relative phase.

One slightly more realistic concern is not with the position perturbation method, but with the fact we only implemented it in judgement tasks (Bingham and Pagano, 1998). Task dynamically, the judgement task is modelled by integrating the coupling term over a 2 s window, which is different from using the coupling term to coordinate two limbs. Evidence does show a close relationship between the judgement and production tasks, however; learning to improve on the judgement task transfers to the production task (Wilson et al., 2010a) and vice versa (e.g., Snapp-Childs et al., 2015; Leach et al., 2021, and the current experiment). The tasks overlap informationally, and that remains the important overlap. That said, it will be important to future work to informationally perturb the entire performance dynamic.

We propose at this stage that in both experiments, participants did learn to use relative position, and that the difference emerges over the *process* of learning. Herth et al. (2021) present an expanded version of the Bingham model that accounts for learning 90° and system behaviour post-learning. They add the option of a second coupling term (normed relative position) and a mechanism for switching between the two couplings. This mechanism simply entails using the most detectable variable, and switching if that variable falls below the current perceptual threshold for that variable.

The critical part for us is how they account for the process of learning. At the beginning, participants only have one coupling option; relative direction. They try to use this coupling to produce 90° (or 60°) but only succeed to a certain extent. In the absence of feedback, participants cannot use relative direction to produce sufficiently stable examples of 90° (or 60°) and so relative position is never available to be learned. With coordination feedback, they are supported as they try to produce 90° (or 60°) via relative direction, relative position becomes available, and is learned because it helps (the need for this feedback support was confirmed by Wilson et al., 2010b).

In ecological task dynamic terms, training does not change limb dynamics, and learning both 60° and 90° entails using relative direction and the feedback to bootstrap their way into moving so that relative position is sufficiently invariant to be learned (c.f., Gibson and Gibson, 1955). So both of these trained systems have access to the same resources. By Herth et al’s analysis, the only remaining dynamical component that might be different between the two systems is the threshold-based mechanism for switching which variable is currently being used.

That mechanism plays out in the correction of errors. If someone is trying to perform 90° using relative position but accidentally slips into doing 0° or 180° using relative direction, the required correction back to 90° entails switching information variables immediately; the threshold for detecting relative direction at 90° is too high, and relative position is the only detectable option. If, however, someone is trying to perform 60° using relative position but accidentally slips into doing 0° using relative direction, the required correction may not mandate an information switch, at least not immediately. The threshold for detecting relative direction at 60° is high but not catastrophically so, and it may take longer before relative position is a clear winner in the competition embodied in the switching mechanism.

If this is the case, then what is being learning is not just a new information coupling, but a way to shift between couplings, and the way that mechanism plays out at 60° suits 30° and 90° but not 120°, while the way that mechanism plays out at 90° suits 60° and 120° but not 30°. The data and simulations from Herth et al. (2021) support this overall analysis, but there is not yet any specific test of this hypothesis and so it remains completely provisional at this time.

### Summary

The current experiment followed on from Leach et al. (2021)’s investigation into learning and transfer of learning. How this works hinges on what constitutes a task; this experiment takes an ecological task dynamical approach to this question in which tasks are defined at the organism-environment scale, with organism and environmental dynamics coupled via perceptual information. While we did not find all of our predicted results, the pattern of data across the two experiments has provided us with a great deal of information about what learning does to the task dynamics of coordinated rhythmic movement. This work continues to be inform and be informed by the mechanistic modelling work by Bingham and colleagues (Bingham, 2001, 2004a,b; Snapp-Childs et al., 2011, 2015; Herth et al., 2021; see Golonka and Wilson, 2012, 2019 for more about the mechanistic aspect) which in turn is informed by and informing the development of the ecological task dynamical approach to perception and action.

## Data Availability Statement

The datasets presented in this study can be found in online repositories. The names of the repository/repositories and accession number(s) can be found below: https://osf.io/qtx8f/.

## Ethics Statement

The studies involving human participants were reviewed and approved by Psychology Ethics Committee, Leeds Beckett University. The patients/participants provided their written informed consent to participate in this study.

## Author Contributions

DL planned and ran the studies, analysed the data, and writing the manuscript. ZK and AW planning the studies and writing the manuscript. All authors contributed to the article and approved the submitted version.

## Conflict of Interest

The authors declare that the research was conducted in the absence of any commercial or financial relationships that could be construed as a potential conflict of interest.

## Publisher’s Note

All claims expressed in this article are solely those of the authors and do not necessarily represent those of their affiliated organizations, or those of the publisher, the editors and the reviewers. Any product that may be evaluated in this article, or claim that may be made by its manufacturer, is not guaranteed or endorsed by the publisher.

## Footnotes

1 Golonka and Wilson (2019) explicitly connect this ecological approach to the neo-mechanist literature in philosophy. The key to being a mechanistic explanation (vs a functional description) is that your model of the task only contains terms that represent the real parts and processes that have been shown to be involved in the task being explained. This is why we emphasise the issue of whether various parts are “real” or not throughout the article.

2 Phase cycles that incur movement of opposing directions (oscillators moving toward or away from one another) incur the presence of relative speed. The more the phase cycle incurs movements of opposing direction, the greater the presence of relative speed. This occurs in a linear fashion; at 0° the motion of both oscillators always remains fixed in the same direction, thus the relative speed at 0° is zero. Relative speed is at its highest throughout the movement cycle of 180°, whereby the motion of both oscillators always remains fixed in the opposite direction (Bingham, 2004b; Wilson et al., 2005; Wilson and Bingham, 2008; Snapp-Childs et al., 2011).

3 We have kept this as an exploratory analysis because it was not included in our preregistration (Leach et al., 2017).

## Appendix 1 – Power Analysis

Two power analyses were performed using G^∗^Power using the results of Leach et al. (2021) as input.

### Detecting Very Large Transfer (Input From 60°)

The following is a power analysis performed on the performance data for detecting transfer from 90° to 60°. The analysis is in the form of a one-sided *t*-test, as is the generation of the *t*-value from the contrast analysis.

**Input:** Tail(s) = One

Effect size *d* = 2.322

α err prob = 0.05

Total sample size = 10

**Output:** Noncentrality parameter δ = 7.3428087

Critical *t* = 1.8331129

Df = 9

Power (1-β err prob) = 0.9999998

Power to observe very large effects of transfer (*d* = 2.322) with *N* = 10 is 1-β of >0.99. This level of power is achieved at *N* > = 6.

### Detecting Large Transfer (Input From 120°)

The following is a power analysis performed on the performance data for detecting transfer from 90° to 120°.

**Input:** Tail(s) = One

Effect size *d* = 1.705

α err prob = 0.05

Total sample size = 10

**Output:** Noncentrality parameter δ = 5.3916834

Critical *t* = 1.8331129

Df = 9

Power (1-β err prob) = 0.9995092

Power to observe an effect size of *d* = 1.705 with the above input is 1-β > 0.99. This level of power is achieved at *N* > = 8.
